# Clinical features, pathogens, and prognosis of immunocompromised host pneumonia in patients with malignancies

**DOI:** 10.3389/fcimb.2025.1646513

**Published:** 2025-11-18

**Authors:** Xiang-Zhi Fang, Zi-Han Liu, Li-Min Duan, Lu Yao, Ji- Qian Xu, Xiao-Bo Yang, Le-Hao Ren, Yong-Xiang Jiang, Sheng-Wen Sun, You Shang, Yin Yuan

**Affiliations:** Department of Critical Care Medicine, Union Hospital, Tongji Medical College, Huazhong University of Science and Technology, Wuhan, China

**Keywords:** pneumonia, patients with malignancies, etiology, prognosis, immunocompromised hosts

## Abstract

**Background:**

Cancer patients face elevated risks of severe pulmonary infections due to malignancy-related immunosuppression and anti-neoplastic therapy. Comprehensive data on the etiology and prognostic factors remain limited.

**Methods:**

This prospective cohort study enrolled 115 patients with malignancies and immunocompromised host pneumonia (ICHP) from July 2023 to July 2024. Pathogens were identified using clinical metagenomics of bronchoalveolar lavage fluid (BALF), supported by CT imaging and clinical evaluation.

**Results:**

Pathogens were detected in 92 patients (80.0%), with 158 potential pathogens detected. Etiologic diagnoses were established by BALF mNGS alone in 68 (73.9%), by combined mNGS plus standard microbiologic testing (SMT) in 24 (26.1%), and by SMT alone in 1 (1.1%). *Pneumocystis jirovecii* (32, 20.3%), SARS-CoV-2 (14, 8.9%), *Aspergillus fumigatus* (13, 8.2%), *Klebsiella pneumoniae* (12, 7.6%) and *Haemophilus influenzae* (10, 6.3%) were the five most common pathogens. Coinfections occurred in 36.5% of all enrolled patients. Death at 28 days, ICU admission, Death at ICU was more frequent among patients with polymicrobial infections than single pathogen infection, though this difference was not statistically significant. Use rate of vasoactive drugs was significantly higher in patients with coinfection than in patients with single-pathogen infection (39.1% vs. 16.0%). invasive mechanical ventilation (IMV) (OR = 22.86, p=0.047), vasopressor use (OR = 72.69, p=0.039), and higher Acute Physiology and Chronic Health Evaluation II (APACHE II) scores (OR = 1.46, p=0.016) were associated with increased 28-day all-cause mortality.

**Conclusion:**

Patients with malignancies and evaluated for pulmonary infection were found to have unique microbiological profiles detected by BAL metagenomic sequencing. Co-detection of potential pathogens was high, and associated with high 28-day all-cause mortality.

## Introduction

Immunocompromised hosts (ICHs), particularly those with malignancies, face substantially elevated risks of pneumonia due to impaired immune defenses (Chen et al., 2021). These patients experience accelerated disease progression and higher mortality than immunocompetent individuals (Azoulay et al., 2015). Enhanced survival stems from advances in early detection, multidisciplinary care models, precision oncology, and novel therapeutics. However, this progress is counterbalanced by heightened infection vulnerability: malignancy-associated immunosuppression and antineoplastic therapies increase susceptibility to primary infections, opportunistic pathogens, and latent pathogen reactivation (Nates et al., 2024). Consequently, infections represent a leading complication in cancer populations, with threefold greater risk of fatal outcomes compared to non-cancer patients (Rolston, 2017; Zheng et al., 2021).

The diagnosis and management of pulmonary infections in ICHs present unique challenges. Compared to immunocompetent patients, immunocompromised host pneumonia (ICHP) exhibits: Higher prevalence of atypical pathogens (Azoulay et al., 2020; Ramirez et al., 2020; Kreitmann et al., 2024), Frequent polymicrobial infections (e.g., viral–viral, viral–fungal, or bacterial–viral co-infection) (Woodhead et al., 2011; Olson and Davis, 2020), Non-specific clinical presentations (e.g., absence of fever in 40% of neutropenic pneumonias). These complexities contribute to high rates of initial antimicrobial failure.

Furthermore, data on ICHP in patients with malignancies are scarce. Despite clinicians widely recognize the heightened risk of lung infections in immunocompromised patients, they are often excluded from pneumonia clinical guidelines and treatment trials (Woodhead et al., 2011; Olson and Davis, 2020; Rello, 2024). Despite inherent limitations in differentiating colonization from infection, clinical metagenomics serves as a promising yet evolving tool for characterizing respiratory microbiota (Chiu and Miller, 2019). In this prospective study, we analyzed 115 patients with malignancies and ICHP using clinical metagenomics to characterize microbial profiles, clinical features, and outcomes, thereby informing empirical antibiotic selection and treatment optimization.

## Methods

### Study design and patients

#### Study design

**Figure 1:**
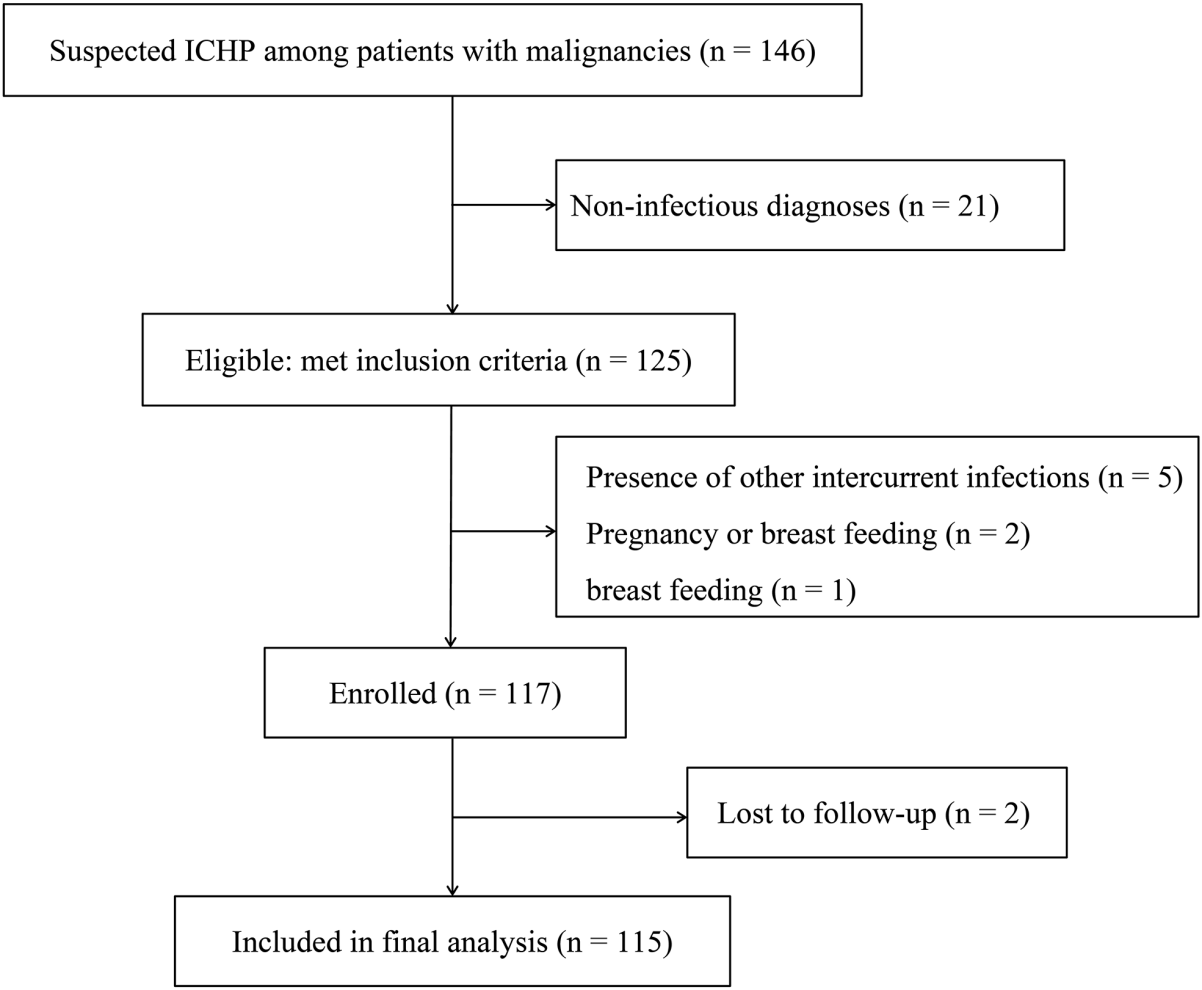
Flow diagram of the study.

Patients with malignancies and suspected pulmonary infections admitted to the oncology ward were prospectively enrolled in a registry between July 2023 and July 2024. This study was carried out in Union Hospital, Tongji Medical College, Huazhong University of Science and Technology. Prior to formal experiments, a research coordinator responsible for recruitment will discuss the research purpose, procedures, and potential risks and benefits with prospective participants. The study flow chart is shown in Figure1. Research protocols were approved by the Research Ethics Committees of Union Hospital, Tongji Medical College, Huazhong University of Science and Technology (0505-01) and registered in Chinese Clinical Trial Registry (MR-42-24-040600).

#### Participants

The inclusion criteria comprised: (1) active malignancy, (2) clinical and radiographic suspicion of pulmonary infection, (3) written informed consent for bronchoscopy, (4) BALF samples meeting technical requirements clinical metagenomics. According to ICHP diagnostic criteria, suspected pulmonary infection was defined by the presence of new or worsening radiographic infiltrates on CT, not necessarily requiring correlative symptoms (Cheng et al., 2023). We defined malignancy in the active stage if it necessitated medical or surgical intervention within the past year or if untreatable metastases were present at the time of inclusion in the study. Given the inherent diagnostic challenges in immunocompromised hosts, all cases represent clinically suspected rather than microbiologically confirmed infections at enrollment.

All participants underwent comprehensive non-infectious evaluation prior to BAL sampling through a standardized two-step protocol:

Step 1: Objective Testing Phase (Completed within 24h of pre-enrollment).

(1) Imaging diagnostics: High-resolution computed tomography (HRCT) for interstitial patterns (UIP/NSIP criteria); CT pulmonary angiography (CTPA) for suspected pulmonary embolism (Wells score > 4). (2) Serum biomarkers: C-reactive protein (CRP) < 10 mg/L; procalcitonin (PCT) < 0.1 ng/mL; rheumatologic serologies (ANA, ANCA, anti-CCP). (3) Microbiological prescreening: Blood cultures and multiplex respiratory virus PCR.

Step 2: Multidisciplinary adjudication (blinded to subsequent BAL results).

Three independent clinicians applied consensus criteria. Cases were adjudicated as non-infectious only when all of the following were met: (1) ≥ 2 objective test abnormalities concordant with a non-infectious process (e.g., HRCT UIP pattern plus positive ANA consistent with ILD; CTPA-confirmed thrombus plus elevated D-dimer consistent with PE). (2) Absence of infection indicators: no sustained fever (>38°C), no purulent sputum (Gram-stain leukocytes < 25/HPF), and no leukocytosis (WBC < 10×10^9^/L). (3) Plausible alternative diagnosis fully explaining radiographic findings.

Patients were excluded if any one of the following criteria was met: (1) under 18 years of age; (2) noninfectious pulmonary interstitial diseases, lung atelectasis, pulmonary embolism, or other diseases leading to related lung changes or symptoms; (3) presence of other intercurrent infections; or (4) pregnancy or breast feeding.

### Sampling, and pathogen detection

All participants underwent chest CT scans before enrollment. Bronchoalveolar lavage fluid (BALF) samples were collected within 48 hours and subjected to metagenomic next-generation sequencing (mNGS). Results are generally available within 36h. We included results from the initial instance of clinical metagenomics when performed multiple times.

Data on prophylactic antimicrobial regimens administered within the 3 months prior to enrollment were systematically collected from electronic medical records. This included the type of prophylaxis (e.g., antibacterial, antifungal, antipneumocystis), drug name, dosage, and duration of administration. Laboratory tests including routine test of blood, liver and kidney function, electrolytes in blood, coagulation, coagulation function (APTT, PT, TT, and D-dimer) after admission were done within 24 hours of study inclusion. Other tests include smear, culture, galactomannan (GM) tests and (1, 3)-beta-D-glucan test (G test) of BALF samples and serum samples, inflammatory factors, peripheral blood lymphocyte subsets were performed according to the physician’s decision.

### mNGS sequencing and analysis

Nucleic acids were extracted with kits designed to minimize host carryover. Pathogen DNA was isolated using the QIAamp^®^ UCP Pathogen DNA Kit, which depletes human DNA. RNA was extracted with the QIAamp UCP Pathogen Mini Kit and treated with Turbo DNase to further reduce host background. cDNA was generated and amplified with the Ovation RNA-Seq System, and libraries were prepared after fragmentation using the Ovation Ultralow System v2. High-throughput sequencing was performed on an Illumina NextSeq 550, producing single-end 75-bp reads.

During bioinformatic processing, low-quality reads were removed with fastp. Reads aligning to the human genome (hg38) were filtered out using the Burrows–Wheeler Aligner (BWA). The remaining microbial reads were classified by alignment to a comprehensive pathogen database using the SNAP aligner.

For pathogen identification, thresholds were defined relative to negative controls. When background reads were present in controls, a minimum reads-per-million (RPM) sample-to-control ratio of 10 was required. When a taxon was absent from controls, the following absolute RPM cutoffs were applied: ≥3 reads for bacteria, mycoplasma, chlamydia, DNA viruses, and fungi, and ≥1 read for the Mycobacterium tuberculosis complex. Final clinical interpretation integrated these molecular findings with patient symptoms, laboratory results, and immune status to assess pathogenicity.

### Identification of detected microorganisms

In our study, the identification of detected microorganisms was collaboratively determined by three clinicians through an integrated evaluation of microbiological data, imaging findings, laboratory findings and clinical symptoms, without blinding. Pathogen attribution was based on the concurrent fulfillment of the following three criteria: 1.NGS quantitative thresholds: bacteria/viruses required a coverage depth >10× that of the background microbiota; fungi required a coverage depth >5× that of other fungal species. 2. clinical-radiological correlation: presence of characteristic symptoms (e.g., purulent sputum suggesting bacterial infection) combined with compatible CT patterns (e.g., ground-glass opacities suggestive of Pneumocystis; cavitary lesions suggestive of Aspergillus). 3.supporting biomarkers: elevated levels of relevant biomarkers (e.g., elevated serum or BALF galactomannan for Aspergillus; elevated CRP and PCT suggesting bacterial infection). 4. When mNGS results conflicted with clinical-radiological findings, Multiplex PCR or PCP PCR were performed.

Patients were classified as *Pneumocystis jirovecii* Pneumonia (PJP) negative if clinical metagenomic testing for *Pneumocystis jirovecii* returned negative results. PJP diagnosis required (Tasaka, 2020): (1) positive clinical metagenomic test for *Pneumocystis jirovecii*, (2) presence of consistent clinical manifestations of PJP, and (3) chest CT scan results consistent with PJP. All patients were given guideline-concordant antibiotics according to the detected microorganisms.

### Data collection and definitions

A uniform case report form was filled out for every episode. Clinical data extracted from the electronic medical record system included patient demographics (age, sex), medical history (underlying diseases, comorbidities, malignancy treatment), vital signs, respiratory parameters (fraction of inspired oxygen, respiratory support), arterial blood gas analyses, laboratory results, pathogen detection, imaging findings, and various clinical scores (APACHE II, SOFA, SMART-COP, PSI, CURB-65) along with clinical outcomes. The clinical outcomes encompassed ICU admission, the requirement for invasive mechanical ventilation (IMV), the use of vasoactive drugs, and both ICU and 28-day all-cause mortality.

The criteria for severe pneumonia were defined as the presence of at least one of the following conditions (Martin-Loeches et al., 2023): (1) mechanical ventilation with endotracheal intubation or (2) vasoactive medication despite aggressive fluid resuscitation for infectious shock, as previously described. Hypoxia was defined as PaO_2_ below 60 mmHg or oxygen saturation under 95% on room air. Septic shock was identified in sepsis patients needing vasopressors use to maintain a mean arterial pressure of at least 65 mmHg, and serum lactate levels over 2 mmol/L (18.02 mg/dL), excluding patients with hypovolemia (Shankar-Hari et al., 2016). Definitions of immunocompromised hosts include: (1) active malignancy or malignancy within the past year (excluding localized skin cancer); (2) receipt of anti-cancer chemotherapy; or (3) receipt of specific immunosuppressant therapy (Ramirez et al., 2020).

We recorded standard microbiologic testing (SMT) within ±48 hours of BALF sampling (sputum culture, BALF culture, blood cultures, pathogen-specific PCR, and antigen assays).

Definition of BALF mNGS–only diagnosis (all criteria required): a) BALF mNGS is positive; b) Within ±48 hours of bronchoalveolar lavage(BAL)sampling, no SMT is positive for the same pathogen (species/genus, or—when the final diagnosis is category-level only—within the same pathogen category/spectrum); c) Per prespecified clinical attribution rules, the detection is adjudicated as etiologic for the current episode (excluding obvious colonization/contamination).

Definition of combined diagnosis (all criteria required): a) BALF mNGS is positive; b) ≥1 SMT is positive for the same pathogen (species/genus preferred; if the final diagnosis is category-level, category/spectrum matching is accepted—for example, “viral pneumonia” matching influenza/RSV/hMPV, etc.); c) Clinical attribution supports the organism as etiologic for the episode.

SMT alone diagnosis: BALF mNGS is negative, while SMT is positive and consistent with clinical attribution (e.g., a nasopharyngeal swab positive for influenza A with compatible symptoms/imaging).

Patients received standard oncology department care and were monitored for 28 days post-diagnosis or until discharge. Patients discharged before 28 days were monitored until Day 28 to record outcomes.

### Statistical analysis

The normality of continuous data was assessed using the Skewness–Kurtosis test. Continuous variables with a normal distribution are presented as mean ± standard deviation, and those with a non-normal distribution as median (interquartile range, IQR). Categorical variables are shown as counts (percentages). Between-group comparisons used Student’s t-test for normally distributed continuous variables, the Mann–Whitney U test for non-normally distributed or ordinal variables, and the chi-squared (χ²) test or Fisher’s exact test, as appropriate. All tests were two-sided with a significance level of P<0.05.

Missing data were handled by mean imputation. Univariable analyses first compared the 28-day survivors and non-survivors; variables with P<0.10 in univariable comparisons and judged clinically relevant were considered candidates for multivariable modelling. Univariable and multivariable logistic regression were then used to identify risk factors for 28-day all-cause mortality. We fitted a mutually adjusted multivariable logistic regression (i.e., candidate covariates entered the model simultaneously so that each effect is estimated conditional on the others), without interaction terms or higher-order terms, to limit degrees of freedom. To address multicollinearity, we calculated variance inflation factors (VIF) for all covariates and required VIF<10. Odds ratios (ORs) with 95% confidence intervals (CIs) were reported. Sample size considerations for modelling. No *a priori* sample-size calculation was undertaken; all eligible patients during the study period were included. We considered the events-per-variable (EPV) heuristic when specifying model complexity. With 32 deaths, the ideal EPV≥10 would permit approximately three degrees of freedom. Given the exploratory aim of risk-factor identification and the need to provide covariate-adjusted estimates for key clinical variables, the multivariable results should be regarded as hypothesis-generating and interpreted with caution. Data analyses and figures were performed using IBM SPSS Statistics, version 25.0 (IBM Corp., Armonk, NY, USA) and R, version 4.3.1 (R Foundation for Statistical Computing, Vienna, Austria).

## Results

### Study population

During the study period, 146 patients with malignancies were evaluated for suspected ICHP, all underwent the standardized two-step non-infectious evaluation prior to BAL sampling. Following adjudication, 21 patients were assigned a non-infectious diagnosis and were excluded before enrollment. The remaining 125 patients met inclusion criteria and proceeded to BAL sampling and downstream analyses. During the study period, 8 eligible patients declined BAL. Among the total sample, 117 (93.6%) met the criteria for enrollment, 2 (1.7%) patients were excluded because of missing data. Eventually, 115 (98.3%) patients were included in the final analysis (**Figure 1**). Lung cancer (79, 68.7%) was the most common type, followed by lymphoma (11, 9.6%) and esophageal cancer (8, 7.0%) (**Supplementary Figure S1**). 100% of our cohort had active malignancy. 91 (79.1%) had received chemotherapy, and 53.0% (61/115) had received additional immunosuppressant therapy within the past year. Overall, 88 (76.5%) were male, and hypertension (29, 25.2%) and chronic heart disease (10, 8.7%) were the most common underlying conditions. Fever, sepsis and hypoxia were found in 61 (53.0%), 52 (45.2%) and 48 (41.7) patients, respectively, on admission; these were the three most common clinical features. The median SOFA score was 2 (IQR, 1–4), and the median APACHE II score was 8 (IQR, 5–12). The other characteristics of the patients with malignancies and pulmonary infections are displayed in **Supplementary Table S1**.

### Pathogens in patients with pneumonia with malignancies

**Figure 2:**
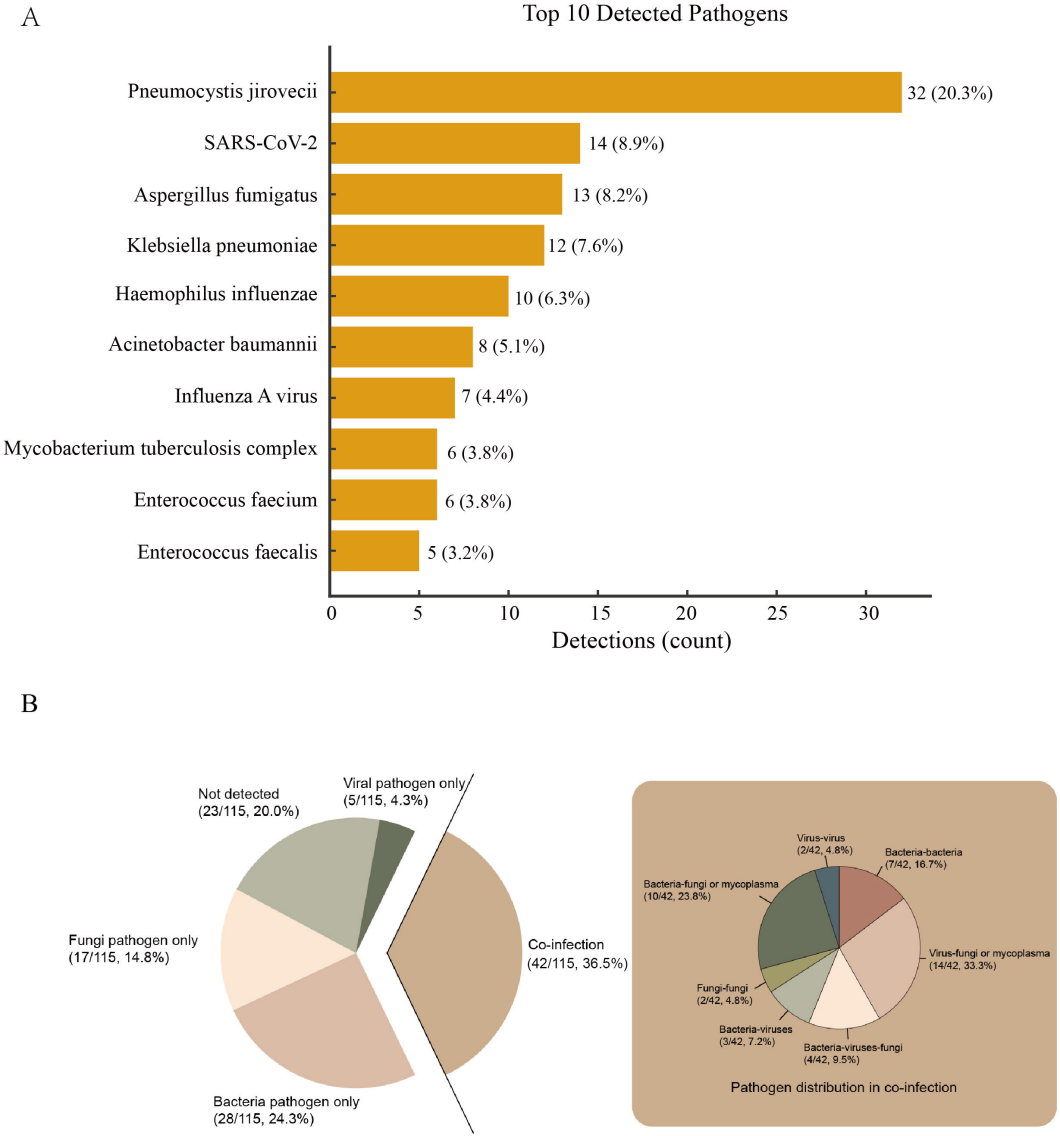
Panel **(A)** Bars denote detection counts; labels indicate count and percentage of all detections (n = 158). Panel **(B)** The big pie chart in the middle shows the proportions of bacterial only, viral only, not detected and co-infection. The small pie chart on the left shows the detailed proportions of co-infection.The central pie chart displays the proportions of bacterial only, viral only, not detected and co-infection. The small pie chart on the left provides a detailed breakdown of the co-infection proportions.

Among the 115 eligible patients, 23 (20.0%) were unable to identify the detected microorganisms. Among the 92 patients with confirmed pathogens, single pathogen infection occurred in 50 (54.3%) and co-infection in 42 (45.7%). Within single-pathogen infections, bacteria accounted for 28 (30.4%), fungi for 17 (18.5%), and viruses for 5 (5.4%). Within co-infections, bacteria–virus occurred in 3 (7.2%), bacteria–fungi or mycoplasma in 10 (23.8%), virus–fungi or mycoplasma in 14 (33.3%), and triple bacteria–virus–fungi in 4 (9.5%) (**Figure 2B**).

A total of 158 pathogens were identified in the 92 patients with confirmed pathogens. The most common potential pathogens detected were *Pneumocystis jirovecii* (32, 20.3%), SARS-CoV-2 (14, 8.9%), *Aspergillus fumigatus* (13, 8.2%), *Klebsiella pneumoniae* (12, 7.6%), *Haemophilus influenzae* (10, 6.3%), *Acinetobacter baumannii* (8, 5.1%), Influenza A virus (7, 4.4%), *Mycobacterium tuberculosis complex* (6, 3.8%), *Enterococcus faecium* (6, 3.8%) and *Enterococcus faecalis* (5, 3.2%) (**Figure 2A**).

### Unique diagnostic contribution of BALF mNGS

Among the 115 patients, 35 (30.4%) underwent blood culture (fever >38.5 °C). 2 (5.7%) blood cultures were positive, both yielding coagulase-negative staphylococci (CoNS). These positives were discordant with BALF mNGS: in case 1, blood cultures grew *Staphylococcus epidermidis* while BALF mNGS identified Haemophilus influenzae and *Acinetobacter baumannii*; in case 2, blood cultures grew *Staphylococcus hominis* while BALF mNGS was negative. The remaining 33 (94.3%) blood cultures were negative. To quantify the unique diagnostic rate of BALF mNGS, we analyzed the 92 patients in whom any potential pathogen was detected. In the overall cohort, all patients received SMT. Among the 92 patients with any detection, etiologic diagnoses were established by BALF mNGS alone in 68 (73.9%), by combined mNGS plus SMT in 24 (26.1%), and by SMT alone in 1 (1.08%) (**Table 1**).

**Table 1:** Diagnostic pathways based on BALF mNGS and standard microbiological testing (SMT).

Diagnostic pathway	Total	Example
BALF mNGS–only diagnosis	68/92(73.9%)	mNGS detected *Pneumocystis jirovecii*, while all SMT was negative
Combined diagnosis (mNGS + SMT)	24/92(26.1%)	mNGS and sputum culture both identified *Pseudomonas aeruginosa*
SMT–only diagnosis (mNGS negative)	1/92 (1.1%)	Nasopharyngeal PCR positive for influenza A, mNGS negative

SMT: standard microbiologic testing, within ±48 hours of BALF sampling (sputum culture, BALF culture, blood cultures, pathogen-specific PCR, and antigen assays).

### Trimethoprim–sulfamethoxazole for PJP prophylaxis

Among 36 patients with *Pneumocystis jirovecii* detected by BALF-mNGS, 32 fulfilled diagnostic criteria for PJP and 6 did not. Prophylactic antimicrobial use was common in our cohort, with 80 (69.6%) of patients receiving at least one form of prophylaxis prior to the onset of pneumonia. 30 (26.1%) of patients were receiving TMP-SMX. 17 (14.8%) of patients received systemic antifungal prophylaxis, most commonly with voriconazole. 12 (10.4%) received antiviral prophylaxis.

### Laboratory findings

Routine blood and biochemistry data were collected from all pneumonia patients with malignancy at admission. The median C-reactive protein concentration was 69.7 mg/L (reference range, < 8.020). The average lymphocyte count was 0.7 × 10^9^/L, which was below the normal range of 1.1–3.2 × 10^9^/L. The Th/Ts (CD4/CD8) ratio decreased to 1.1, which was below the reference range of 1.4–2.0. As anticipated, human IL-6 levels rose to an average of 53.1 pg/ml, exceeding the reference range of ≤20.0 pg/ml. Other laboratory findings are displayed in **Supplementary Table S1**.

### Clinical outcomes

**Figure 3:**
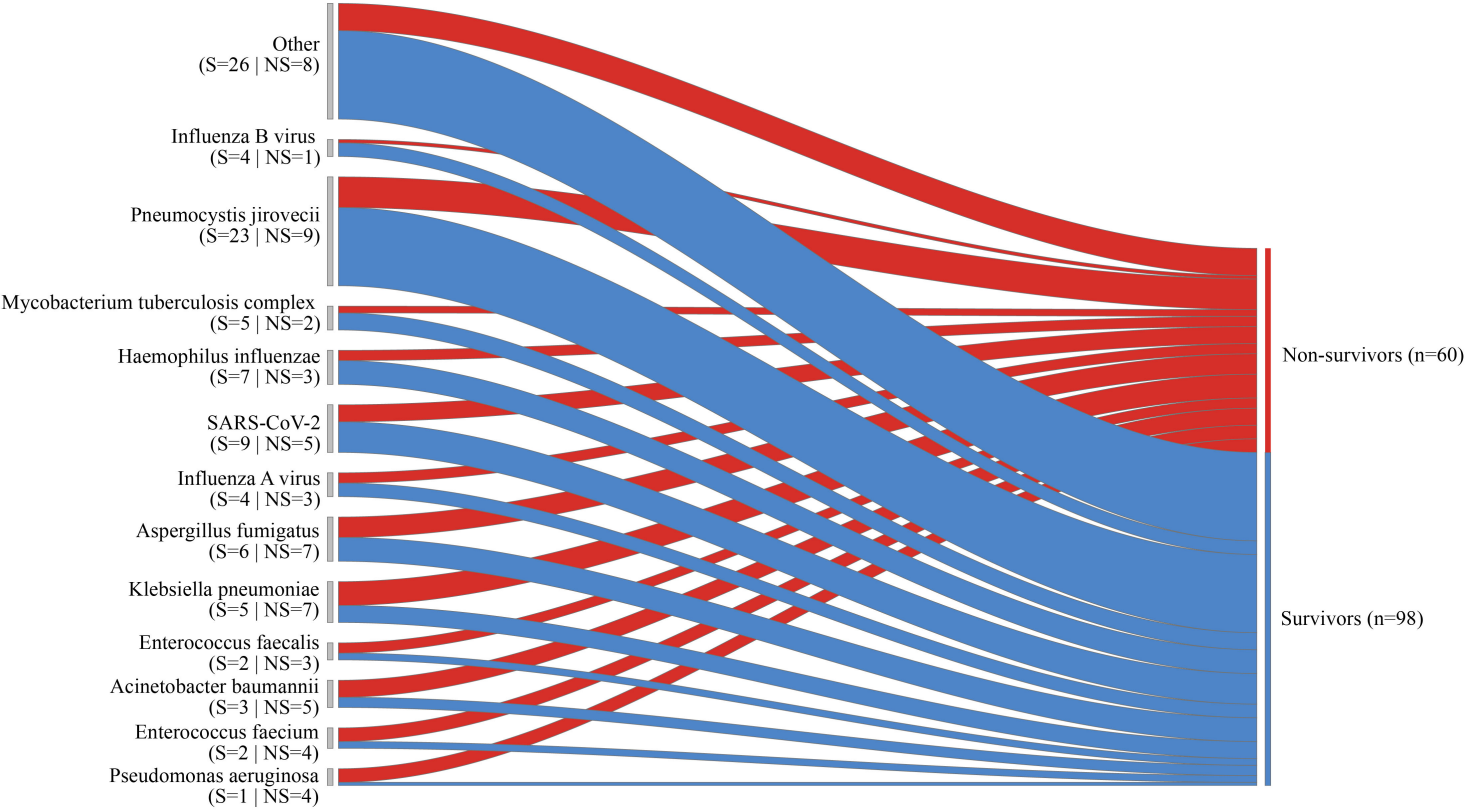
The Sankey diagram depicts the diverse outcomes for specific pathogens, with the width of the arrows representing the proportional flow rate of cases, categorized as survivors (indicated in blue) or non-survivors (indicated in red). The numbers displayed above the arrows correspond to the number of cases.

Among the 115 patients included in the final analysis, the 28-day all-cause mortality was 32 (27.8%) (**Table 2**). The 28-day all-cause mortality was significantly higher in pathogen-positive patients than in pathogen negative patients (33.7% vs 4.3%, p=0.005) (**Table 2**, **Supplementary Table S3**). The 28-day all-cause mortality was more frequent among patients with polymicrobial infections than single pathogen infection (40.5% vs 28.0%, p=0.207), though this difference was not statistically significant (**Table 2**, **Supplementary Table S4**). The 28-day all-cause mortality of patients with *Pseudomonas aeruginosa*, *Enterococcus faecium* and *Acinetobacter baumannii* were 80.0%, 66.7% and 62.5%, respectively (**Figure 3**).

**Table 2:** Clinical outcomes.

Patient group	Total	Pathogen negative	Pathogen positive	Single pathogen infection	Coinfected	Bacteria infection	Fungi infection	Viruses infection	Bacteria-fungi or mycoplasma coinfected	Virus - fungi or mycoplasma coinfected	Bacteria-bacteria coinfected
ICU admission	34/115 (29.6%)	4/23 (17.4%)	30/92 (32.6%)	13/50 (26.0%)	17/42 (40.5%)	10/28 (35.7%)	3/17 (17.6%)	0	5/10 (50.0%)	5/14 (35.7%)	2/7 (28.6%)
ICU mortality	25/34 (73.5%)	1/4 (25.0%)	24/30 (80%)	10/13 (76.9%)	14/17 (82.4%)	7/10 (70%)	3/3 (100%)	0	3/5 (60.0%)	4/5 (80.0%)	2/2 (100%)
28-day mortality	32/115 (27.8%)	1/23 (4.3%)	31/92 (33.7%)	14/50 (28.0%)	17/42 (40.5%)	9/28 (32.1%)	4/17 (23.5%)	1/5 (20.0%)	3/10 (30.0%)	4/14 (28.6%)	3/7 (42.9%)
Vasoactive drugs	18/115 (15.7%)	1/23 (4.3%)	17/92 (18.5%)	8/50 (16.0%)	9/42 (21.4%)	6/28 (21.4%)	2/17 (11.8%)	0	0	4/14 (28.6%)	2/7 (28.6%)
IMV	31/115 (27.0%)	1/23 (4.3%)	30/92 (32.6%)	11/50 (22.0%)	15/42 (35.7%)	9/28 (32.1%)	2/17 (11.8%)	0	5/10 (50.0%)	4/10 (40.0%)	1/7 (14.3%)

IMV, invasive mechanical ventilation; ICU, intensive care unit.

Among the patient cohort, 34 (29.6%) necessitated admission to the ICU. The mortality rate within the ICU was 73.5%. Among patients with single pathogen infection, 13 (26%) required ICU admission, and the ICU mortality rate was 76.9%. Among patients with polymicrobial infections, 17 (40.5%) required ICU admission, and the ICU mortality rate was 82.4% (**Table 2**). Patients with co-infection required IMV significantly more than patients with single pathogen infection (35.7% vs. 22.0%, p=0.428), though this difference was not statistically significant. (**Supplementary Table S4**).

### Independent predictor of 28-day all-cause mortality

**Figure 4:**
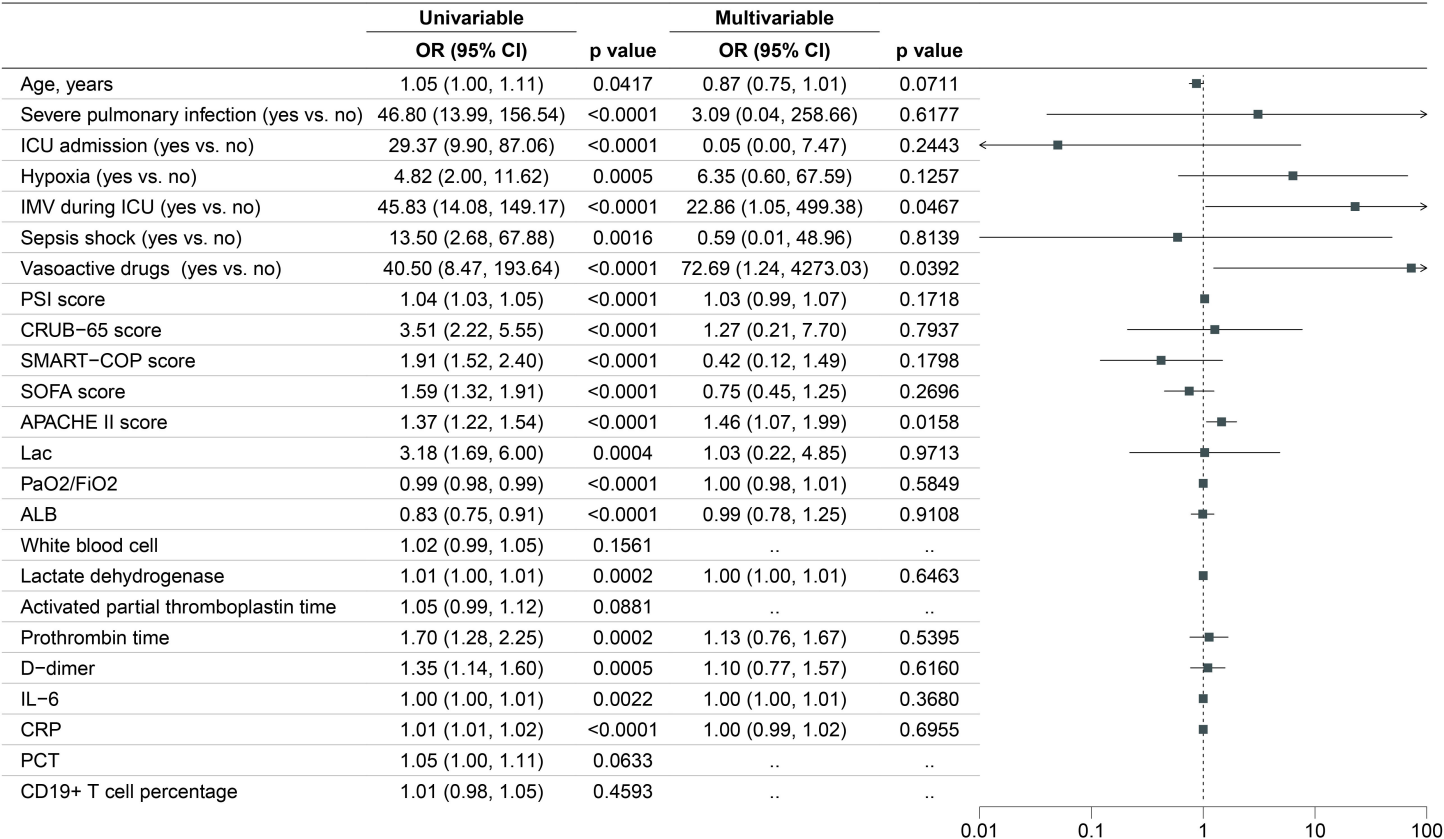
Risk factors associated with 28-day all-cause mortality.

Significant differences across various variables were noted between the 28-day survivor and non-survivor groups (**Supplementary Table S1**). In our univariate logistic regression analysis, we examined variables including age, severe pulmonary infection, ICU admission, needed IMV, hypoxia, septic shock, vasoactive drugs, PSI score, CRUB-65 score, SMART-COP score, SOFA score, APACHE II score, Lac, PaO2/FiO2, ALB, white blood cell, lactate dehydrogenase, activated partial thromboplastin time, prothrombin time, D-dimer, IL-6, CRP, PCT, CD19+ T cell percentage. Significant differences were observed for most variables, except for white blood cell, activated partial thromboplastin time, PCT and CD19+ T cell percentage. We subsequently included the variables with P-value less than 0.05 for the multivariable logistic regression model. **Figure 4** displayed the results of the univariable and multivariable logistic regression models. The results of the multivariate logistic regression are shown on the right with a forest plot. The independent risk factors for 28-day all-cause mortality were needed IMV (OR, 22.86; 95% CI, 1.05-499.38; p =0.0467), used vasoactive drugs (OR, 72.69; 95% CI, 1.24-4273.03; p =0.0392) and higher APACHE II score (OR, 1.46; 95% CI, 1.07, 1.99; p=0.0158).

## Discussion

In this prospective real-world cohort of oncology patients undergoing BAL for suspected pneumonia, we evaluated the pathogen detection yield of BALF mNGS versus conventional testing. The etiology varies and includes bacteria, viruses, and fungi. Etiologic agents such as *Pneumocystis jirovecii*, *Aspergillus* and *K. pneumoniae* are distinctly uncommon in pneumonia patients but relatively rare in patients with malignancies and pneumonia.

Early identification of uncommon pathogens enables physicians to promptly switch to effective targeted treatment. Our study revealed a greater proportion of patients with identified pathogens, with a distribution distinct from that reported in previous studies (Fernández-Cruz et al., 2020; Wang et al., 2020). This variation is likely due to the use of clinical metagenomics, which are known for their higher sensitivity (Sun et al., 2021). Additionally, in this study, lower respiratory samples are collected in all patients for microbial detection, which is recommended for detecting pathogens in pneumonia patients, particularly those who are immunocompromised and hospitalized (Murdoch, 2016). These might together contribute to the different results in this study compared with those in other studies.

Our findings are in accordance with previous findings reporting a significantly greater proportion of coinfections and fungal infections in patients with malignancies than in the immunocompetent population (Fernández-Cruz et al., 2020; Wu, 2023; Garnacho-Montero et al., 2024). These results revealed the complexity of the pathogen in patients with malignancies and pulmonary infection, which increases the difficulty of choosing the initial antibiotic. Interestingly, the significantly higher mortality observed in coinfected patients underscores the clinical challenge posed by polymicrobial infections. This may stem from delayed appropriate antimicrobial coverage, synergistic pathogen interactions, or compounded immune dysregulation.

*Pneumocystis jirovecii* commonly occurs in immunocompromised individuals, including those with solid or hematological cancers undergoing chemotherapy, which is consistent with previous research findings (Waks et al., 2015; Takeda et al., 2021).The high prevalence of *Pneumocystis jirovecii* in our cohort underscores its role as a critical opportunistic pathogen in immunocompromised hosts. However, distinguishing true infection from colonization remains challenging. Our stringent diagnostic criteria align with recent guidelines recommending a multi-modal approach to avoid overdiagnosis (Tasaka, 2020). *Pneumocystis jirovecii* patients received TMP-SMX within 48 hours of diagnosis, which may explain their favorable survival. Further research is needed to comprehend the epidemiology of severe pneumonia caused by *Pneumocystis jirovecii*, particularly through the use of nucleic acid amplification tests.

Enrollment in this study required both clinical indication for and patient consent to undergo bronchoscopy with BAL. This inherently introduces a selection bias, as it excludes: (i) the most critically ill or hemodynamically unstable patients for whom invasive procedures were deemed too high-risk; (ii) patients with contraindications to bronchoscopy (e.g., severe coagulopathy, refractory hypoxemia); Consequently, our cohort likely represents a more clinically stable subgroup of immunocompromised patients with pneumonia, potentially limiting the generalizability of our findings to the most severe cases in the ICU. our findings must be interpreted in the context of a significant selection bias introduced by the requirement for BAL. As confirmed by our analysis, the study cohort had lower illness severity and mortality than eligible non-participants. Therefore, the microbial ecology and outcomes described here may not be fully generalizable to the most critically ill immunocompromised patients in the intensive care unit, in whom the pathogen profile may be different and the burden of organ failure greater. The performance and utility of BALF-mNGS in this excluded, high-acuity population remain an important area for future investigation.

Limited research has focused on identifying mortality risk factors in cancer patients with pneumonia. Multivariate analysis identified three independent predictors of 28-day all-cause mortality: requirement for IMV, vasopressor use, and higher APACHE II scores. The APACHE II system, used globally in ICUs since 1985, assesses disease severity and predicts hospital mortality by evaluating 12 physiological parameters, chronic health status, and age (Ho et al., 2005; Chen et al., 2007; Frost et al., 2009; Lee et al., 2010). Similarly, the administration of vasopressor drugs and the requirement for IMV indicate that the patient may be experiencing circulatory or respiratory failure, which is associating to a higher risk of death. Likewise, previous studies have emphasized the severity of a longer duration of initial IMV, which deserves further attention from clinicians (Van Den Akker et al., 2013; Dou et al., 2023). Furthermore, Roberts et al. reported in a multicenter prospective cohort study between September 2017 and February 2018 that increasing vasopressor dosing intensity during the first 24 hours after septic shock was associated with increased mortality (Roberts et al., 2020).

Our study has several limitations. First, as it was performed in a single center, our results may not necessarily be extrapolated to other institutions. Thus, larger multicenter studies are necessary. Second, although mNGS provides unparalleled breadth in microbial detection, its clinical interpretation is fraught with the challenge of differentiating true infection from colonization or environmental contamination. This is a fundamental limitation of all nucleic acid-based detection methods and is particularly acute in the respiratory tract, a non-sterile site with a complex microbiome. Our stringent criteria for pathogen attribution aimed to mitigate this but cannot eliminate the uncertainty. Furthermore, while mNGS demonstrates high analytical sensitivity for a wide range of pathogens, its clinical sensitivity and specificity vary considerably compared to gold-standard diagnostic methods. False-positive results can arise from environmental contamination, database errors, or background nucleic acid from colonizing flora. Conversely, false-negative results may occur due to low pathogen biomass, inefficient nucleic acid extraction, or overwhelming host DNA. These performance characteristics, highlighted in previous studies, mean that mNGS findings must be interpreted as a piece of the diagnostic puzzle and always correlated rigorously with clinical and radiological findings. Third, immunocompromised people are often excluded from pneumonia clinical guidelines and treatment trials. Despite the ICHP diagnostic criteria issued by the American Thoracic Society, the diagnosis of lung infection in patients with malignancies remains challenging.

## Conclusion

In this prospective cohort of immunocompromised patients with suspected pneumonia, BALF metagenomic sequencing demonstrated significant potential for pathogen detection, revealing complex microbial profiles with frequent co-detections that were associated with poor outcomes. While this methodology provides valuable diagnostic information that may complement conventional approaches, our findings indicate that mNGS results require careful clinical correlation to differentiate between colonization and infection. Further validation is needed to fully establish the clinical utility of BALF-mNGS in guiding management decisions for this vulnerable population.

## Data Availability

The raw data supporting the conclusions of this article will be made available by the authors, without undue reservation.

## Supplementary material

Supplementary Table 1 Demographic, Clinical, and Radiological Characteristics Stratified by 28-day Survival.

Supplementary Table 2 BALF mNGS detections with their frequencies and the final clinical adjudication.

Supplementary Table 3 Comparison between patients with pathogen negative and pathogen positive.

Supplementary Table 4 Comparison between patients with Single pathogen infection and Coinfected.

Supplementary Figure 1 Underlying cancer in pneumonia episodes.
