# Supplementary material for: Clinical features, pathogens, and prognosis of immunocompromised host pneumonia in patients with malignancies
Source: Front Cell Infect Microbiol. 2025 Nov 18;15:1646513. doi: 10.3389/fcimb.2025.1646513 (PMC12669106; doi:10.3389/fcimb.2025.1646513)
Supplement: Supplementary Table 1 — Demographic, Clinical, and Radiological Characteristics Stratified by 28-day Survival. [file Table1.docx]

| Supplementary Table S1. Demographic, Clinical, and Radiological Characteristics Stratified by 28-day Survival | | | | |
| --- | --- | --- | --- | --- |
| **Variables** | **Total** | **Survival** | **Non-survivors** | **p** |
|  | **(n = 115)** | **(n=83)** | **(n = 32)** |  |
| **Age (years, Mean ± SD)** | 61.7 ± 9.0 | 60.7 ± 8.8 | 64.5 ± 9.1 | 0.043 |
| **BMI (median, IQR)** | 22.8 ± 3.4 | 23.0 ± 3.5 | 22.1 ± 3.2 | 0.185 |
| **Male (n, %)** | 88 (76.5%) | 62 (74.6%) | 26 (81.3%) | 0.619 |
| **Solid tumor (n, %)** | 104 (90.4%) | 75 (90.4%) | 29 (90.6%) | 1 |
| **Recent Therapy (past year) (n, %)** | | | | |
| Recent chemotherapy | 91 (79.1%) | 69 (83.1%) | 22 (68.8%) | 0.148 |
| Recent immunodepressant | 61 (53.0%) | 45 (54.2%) | 16 (50.0%) | 0.843 |
| **Clinical Features (n, %)** | | | | |
| Fever | 61 (53.0%) | 38 (45.7%) | 23 (71.9%) | 0.021 |
| Diarrhoea | 1 (0.9%) | 0 (0%) | 1 (3.1%) | 0.278 |
| Hypoxia | 48 (41.7) | 26 (31.3%) | 22 (68.8%) | < 0.001 |
| Severe pulmonary infection | 29 (25.2%) | 5 (6.0%) | 24 (75.0%) | < 0.001 |
| Sepsis | 52 (45.2%) | 29 (35.0%) | 23 (71.9%) | < 0.001 |
| Septic shock | 10 (8.7%) | 2 (2.4%) | 8 (25.0%) | < 0.001 |
| ICU admission | 34 (29.6%) | 9 (10.8%) | 25 (78.1%) | < 0.001 |
| IMV during ICU | 31 (26.9%) | 6 (7.3%) | 25 (78.1%) | < 0.001 |
| Vasoactive drugs | 18 (15.7%) | 2 (2.4%) | 16 (50.0%) | < 0.001 |
| **CT Findings (n, %)** | | | | |
| Consolidation | 54 (47.0%) | 34 (41.0%) | 20 (62.5) | 0.062 |
| Ground-glass opacity | 71 (61.7%) | 55 (66.3%) | 16 (50.0%) | 0.163 |
| Pleural effusion | 61 (53.0%) | 35 (42.2%) | 26 (81.3%) | < 0.001 |
| **Any underlying Disease (n, %)** | | | | |
| COPD | 4 (3.5%) | 3 (3.6%) | 1 (3.1%) | 1 |
| Hypertension | 29 (25.2%) | 20 (24.1%) | 9 (28.1%) | 0.837 |
| Chronic heart disease | 10 (8.7%) | 5 (6.0%) | 5 (15.6%) | 0.138 |
| Diabetes Mellitus | 11 (9.6%) | 7 (8.4%) | 4 (12.5%) | 0.496 |
| **Severity Scores** | | | | |
| PSI score (median, IQR) | 70.0 (40.0, 109.0) | 50.0 (40.0, 85.0) | 140.0 (97.5, 160.0) | < 0.001 |
| CRUB-65 score (median, Q1,Q3) | 1.0 (0, 2.0) | 0(0, 1.0) | 3.0 (1.3, 3.0) | < 0.001 |
| SMART-COP score (median, IQR) | 2.0 (1.0, 5.0) | 1.0 (1.0, 3.0) | 7.0 (4.0, 8.0) | < 0.001 |
| SOFA score (median, IQR) | 2.0 (1.0, 4.0) | 1.0 (1.0, 2.0) | 8.0 (3.0, 13.0) | < 0.001 |
| APACHE II score (median, IQR) | 8.0 (5.0, 12.0) | 7.0 (4.0, 8.5) | 19 (11.8, 23.3) | < 0.001 |
| PaO2/FiO2<300 (n, %) | 35 (30.4%) | 11 (13.3%) | 24 (75.0%) | < 0.001 |
| **Laboratory Findings** | | | | |
| PH | 7.43 (7.4, 7.45) | 7.43 (7.41, 7.45) | 7.4 (7.28, 7.44) | 0.02 |
| PCO2 | 34.7 (30.8, 38.7) | 34.5 (30.55, 37.95) | 36.8 (32.68, 42.8) | 0.032 |
| PaO2/FiO2 (mmHg, median, IQR) | 346.4 (260.0, 404.0) | 367.0 (310.0, 420.0) | 233.0 (131.5, 315.0) | < 0.001 |
| Lactate (mmol/L, median, IQR) | 1.0 (0.7, 1.4) | 0.9 (0.7, 1.2) | 1.35 (1.0, 2.4) | < 0.001 |
| RBC | 3.4 ± 0.82 | 3.57 ± 0.74 | 2.96 ± 0.86 | < 0.001 |
| Hb | 105 (89.5, 120.5) | 110 (97, 122) | 89.5 (70.5, 101.5) | < 0.001 |
| Hct | 32.2 (27.2, 36.9) | 33.9 (29.5, 37.2) | 27.7 (21.5, 32.2) | < 0.001 |
| Platelet (×109/L, Mean ± SD) | 221.5 ± 110.8 | 232.01 ± 98.1 | 194.2 ± 136.5 | 0.159 |
| White blood cell count (×109/L, median, IQR) | 6.3 (4.1, 9.5) | 5.56 (3.8, 8.1) | 9.3 (5.6, 16.4) | < 0.001 |
| Neutrophil count (×109/L, median, IQR) | 5.2 (3.0, 8.0) | 3.9 (2.8, 6.4) | 8.3 (4.5, 15.2) | < 0.001 |
| Lymphocytes (×109/L, median, IQR) | 0.7 (0.4, 1.1) | 0.78 (0.5, 1.1) | 0.58 (0.4, 1.0) | 0.303 |
| Activated partial thromboplastin time (s, median, IQR) | 36.5 (33.4, 39.4) | 36.4 (32.4, 39.2) | 37.45 (34.7, 40.2) | 0.093 |
| Prothrombin time (s, median, IQR) | 13.5 (12.9, 14.7) | 13.2 (12.7, 13.9) | 15.4 (14.4, 16.7) | < 0.001 |
| Fibrinogen (g/L, Mean ± SD) | 5.4 ± 1.9 | 5.35 ± 1.7 | 5.4 ± 2.3 | 0.953 |
| D-dimer (mg/L, median, IQR) | 1.5 (0.8, 2.4) | 1.1 (0.6, 1.5) | 2.5 (1.9, 8.0) | < 0.001 |
| Albumin (g/L, Mean ± SD) | 34.5 ± 6.1 | 36.0 ± 6.0 | 30.6 ± 4.3 | < 0.001 |
| Serum creatinine (μmol/L, median, IQR) | 62.7 (51.4, 82.1) | 64.7 (53.9, 80.7) | 54.8 (43.9, 81.3) | 0.113 |
| Lactate dehydrogenase (IU/L,median, IQR) | 239.5 (167.5, 329.5) | 213 (161.5, 275) | 346 (239.5, 458.75) | < 0.001 |
| Human IL-6 (pg/ml,median, IQR) | 53.1 (15.6, 96.0) | 52.2 (9.6, 73.1) | 82.0 (53.1, 514.9) | < 0.001 |
| C-reactive protein (mg/L,median, IQR) | 69.7 (17.3, 124.6) | 57.01 (10.5, 100.7) | 123.7 (58.0, 216.8) | < 0.001 |
| Procalcitonin (ug/L,median, IQR) | 0.1 (0.0, 0.7) | 0 (0, 0.2) | 2.5 (0.1, 5.1) | < 0.001 |
| CD3+ T cell percentage (%,median, IQR) | 71.6 (62.2, 78.0) | 71.6 (61.6, 78.7) | 71.6 (62.7, 75.5) | 0.781 |
| CD3 + CD4+ T cell percentage (%,median, IQR) | 33.7 (26.7, 39.4) | 33.7 (26.3, 38.5) | 33.7 (28.5, 41.6) | 0.578 |
| CD3 + CD8+ T cell percentage (%,median, IQR) | 29.1 (22.6, 35.4) | 29.1 (22.6, 36.2) | 29.1 (23.8, 32.8) | 0.8 |
| CD19+ T cell percentage (%,median, IQR) | 9.5 (4.7, 14.5) | 9.5 (4.5, 14.3) | 11.6 (8.6, 16.8) | 0.082 |
| CD16+CD56+CD3-NK cell percentage (%,median, IQR) | 14.7 (10.2, 21.4) | 14.7 (10.9, 24.8) | 14.7 (8.5, 16.5) | 0.178 |
| Th/Ts（CD4+/CD8+) (%,median, IQR) | 1.1 (0.8, 1.6) | 1.1 (0.9, 1.5) | 1.2 (0.8, 1.6) | 0.47 |

IQR,inter-quartile range. COPD, chronic obstructive pulmonary disease. CURB-65 severity score, measures confusion, urea, respiratory rate, and blood pressure at age 65 years or older. Pneumonia severity index, is based on sex, age, nursing home status, mental status, heart rate, respiratory rate, blood pressure, temperature, selected underlying medical conditions, laboratory values, and the presence or absence of pleural effusion. APACHE II, acute physiology and chronic health evaluation scoring system. SOFA, sequential organ failure assessment;PaO2/FiO2, the ratio of arterial oxygen partial pressure to fractional inspired oxygen.
