# Supplementary material for: Clinical features, pathogens, and prognosis of immunocompromised host pneumonia in patients with malignancies
Source: Front Cell Infect Microbiol. 2025 Nov 18;15:1646513. doi: 10.3389/fcimb.2025.1646513 (PMC12669106; doi:10.3389/fcimb.2025.1646513)
Supplement: Supplementary Table 2 — BALF mNGS detections with their frequencies and the final clinical adjudication. [file Table2.docx]

| Supplementary Table S2. BALF mNGS detections with their frequencies and the final clinical adjudication. | | |
| --- | --- | --- |
| **Cases** | **Detected microorganisms** | **potential pathogen** |
| case 1 | Pneumocystis jirovecii | Streptococcus mitis__145017 Streptococcus oralis__58479 Brevundimonas diminuta__9395 Haemophilus parainfluenzae__4953 Pneumocystis jirovecii__64413 Human herpesvirus 4__276681 |
| case2 | Mycobacterium tuberculosis complex | Mycobacterium tuberculosis complex __846284 |
| case3 | Pneumocystis jirovecii | Streptococcus mitis__230662 Brevundimonas diminuta__132629 Haemophilus parainfluenzae__4680 Pneumocystis jirovecii__1623 Human herpesvirus 4__719448 Human herpesvirus 7__11279 Human herpesvirus 1__246 |
| case4 | Human metapneumovirus | Human metapneumovirus__2416 Human rhinovirus C__1542 Human herpesvirus 7__5849 |
| case5 | Acinetobacter pittii | Acinetobacter pittii__2449 Human herpesvirus 7__17434 |
| case6 | Staphylococcus aureus  Legionella longbeachae | Staphylococcus aureus__1503 Legionella longbeachae__108 |
| case7 | （-） | Streptococcus mitis__6734 Brevundimonas diminuta__6740 Staphylococcus epidermidis__2898 Human herpesvirus 7__2399 Pneumocystis jirovecii__2 |
| case8 | Pneumocystis jirovecii | Pneumocystis jirovecii__4713 |
| case9 | Aspergillus fumigatus  Aspergillus niger   Aspergillus terreus  Human metapneumovirus | Streptococcus mitis__24034 Haemophilus parainfluenzae__2146 Aspergillus fumigatus__2421 Aspergillus niger__455 Aspergillus terreus__166 Human metapneumovirus__1528191 Human herpesvirus 7__1424 |
| case10 | Klebsiella pneumoniae | Klebsiella pneumoniae__2206 Streptococcus mitis__76903 Streptococcus anginosus__21070 Brevundimonas diminuta__19334 Haemophilus parainfluenzae__8291 Ligilactobacillus salivarius__1078 Human herpesvirus 7__6233 |
| case11 | Mycobacterium tuberculosis complex  Acinetobacter baumannii  Enterococcus faecalis  Klebsiella variicola  Enterobacter cloacae complex  Klebsiella pneumoniae  Rhizopus arrhizus | Mycobacterium tuberculosis complex __868641 Acinetobacter baumannii__8209 Enterococcus faecalis__7246 Klebsiella variicola__317 Enterobacter cloacae complex__3415 Klebsiella pneumoniae__1113 Streptococcus anginosus__387243 Streptococcus mitis__381685 Brevundimonas diminuta__83957 Haemophilus haemolyticus__5019 Haemophilus parainfluenzae__1450 Saccharomyces cerevisiae__45594 Candida albicans__209065 Rhizopus arrhizus__10099 Candida krusei__684 Clavispora lusitaniae__295 Nakaseomyces glabrata__24 Human herpesvirus 1__4194 Human herpesvirus 4__5864 |
| case12 | Pseudomonas aeruginosa Acinetobacter baumannii  Klebsiella pneumoniae | Pseudomonas aeruginosa__81221 Acinetobacter baumannii__1989 Klebsiella pneumoniae__1359 Streptococcus mitis__119991 Streptococcus oralis__19276 Staphylococcus epidermidis__5248 Haemophilus parainfluenzae__568 Human parainfluenza virus type 3__788 Human herpesvirus 5__3977 |
| case13 | Pneumocystis jirovecii Aspergillus fumigatus | Streptococcus mitis__13991 Streptococcus anginosus__2291 Streptococcus oralis__1737 streptococcus constellatus__1432 Brevundimonas diminuta__1125 Pneumocystis jirovecii__28714 Aspergillus fumigatus__266 GB virus C__738 Human herpesvirus 4__775 |
| case14 | Klebsiella pneumoniae | Klebsiella pneumoniae__3279 Staphylococcus hominis__2565 Staphylococcus epidermidis__232 |
| case15 | Mycobacterium tuberculosis complex  Pneumocystis jirovecii | Mycobacterium tuberculosis complex __89 Pneumocystis jirovecii__3126126 Human herpesvirus 7__4871 |
| case16 | （-） | Corynebacterium striatum__1030 Human herpesvirus 7__2962 Human herpesvirus 5__13740 Pneumocystis jirovecii__3 |
| case17 | Pneumocystis jirovecii Human parainfluenza virus type 1 | Staphylococcus epidermidis__1246 Pneumocystis jirovecii__20960 Human parainfluenza virus type 1__3104 |
| case18 | （-） | Human herpesvirus 7__21172 Pneumocystis jirovecii__2 |
| case19 | Pneumocystis jirovecii | Streptococcus mitis__66658 Pneumocystis jirovecii__4191 Human herpesvirus 5__161859 Human herpesvirus 7__5776 Human herpesvirus 1__1929 |
| case20 | Staphylococcus aureus  Pneumocystis jirovecii | Staphylococcus aureus__5235 Streptococcus anginosus__210464 Streptococcus mitis__152056 Abiotrophia defectiva__95907 Haemophilus parainfluenzae__4398 Pneumocystis jirovecii__3347 Human herpesvirus 7__244435 |
| case21 | Enterococcus faecium  Acinetobacter baumannii  Klebsiella pneumoniae  Aspergillus fumigatus  Candida tropicalis  Human coronavirus 229E | Enterococcus faecium__485276 Acinetobacter baumannii__17542 Burkholderia multivorans__7000 Klebsiella pneumoniae__1951 Streptococcus oralis__489552 Staphylococcus epidermidis__9479 Candida albicans__24280 Aspergillus fumigatus__846 Candida tropicalis__1132 Human coronavirus 229E__58400 Human herpesvirus 4__814094 Human herpesvirus 7__63821 Human herpesvirus 1__6773 Human herpesvirus 5__4352 |
| case22 | Pneumocystis jirovecii  SARS-CoV-2 | Serratia marcescens__270 Streptococcus mitis__1148216 Haemophilus parainfluenzae__3937 Staphylococcus hominis__3064 Pneumocystis jirovecii__56379 SARS-CoV-2__25644 Human herpesvirus 7__18084 Human herpesvirus 1__73971 |
| case23 | （-） | Pneumocystis jirovecii__5 |
| case24 | Enterococcus faecium | Streptococcus oralis__4839 Staphylococcus hominis__1449 Enterococcus faecium__4102 Human herpesvirus 5__754 |
| case25 | Enterococcus faecium  Aspergillus fumigatus  Aspergillus flavus  Pneumocystis jirovecii  SARS-CoV-2 | Enterococcus faecium__3990 Abiotrophia defectiva__15467 Aspergillus fumigatus__52034 Aspergillus flavus__18234 Candida albicans__4787 Pneumocystis jirovecii__4692 SARS-CoV-2__1251521 |
| case26 | Aspergillus fumigatus  Human coronavirus 229E | Moraxella catarrhalis__1004057 Streptococcus anginosus__17320 Staphylococcus epidermidis__1961 Aspergillus fumigatus__914 Human coronavirus 229E__237 Human herpesvirus 7__1747 |
| case27 | （-） | （-） |
| case28 | Aspergillus fumigatus  SARS-CoV-2  Influenza A virus | Staphylococcus epidermidis__1426 Aspergillus fumigatus__198 SARS-CoV-2__5039 Influenza A virus__497506 |
| case29 | Haemophilus influenzae | Haemophilus influenzae__102898 Bacteroides fragilis__230 Streptococcus oralis__64244 streptococcus constellatus__30665 Brevundimonas diminuta__30338 Streptococcus mitis__29450 Streptococcus intermedius__20059 Haemophilus parainfluenzae__13132 Streptococcus anginosus__10118 Candida albicans__72 Human herpesvirus 7__24603 Pneumocystis jirovecii__2 |
| case30 | （-） | （-） |
| case31 | （-） | Streptococcus oralis__2849 Human herpesvirus 7__2903 |
| case32 | Pneumocystis jirovecii | Pneumocystis jirovecii__5637 Human herpesvirus 1__73971 |
| case33 | Pneumocystis jirovecii | Moraxella catarrhalis__882658 Pneumocystis jirovecii__6798 |
| case34 | （-） | Streptococcus mitis__2213 Human herpesvirus 6B__50 Human herpesvirus 7__12265 |
| case35 | Aspergillus fumigatus  Aspergillus flavus SARS-CoV-2 | SARS-CoV-2__36977 Human coronavirus 229E__70119 Human herpesvirus 7__748870 Human herpesvirus 4__2313 Aspergillus fumigatus__954 Aspergillus flavus__201 |
| case36 | Klebsiella pneumoniae | Candida albicans__3852 Klebsiella pneumoniae__357872 Human herpesvirus 1__141376 Human herpesvirus 4__8331 |
| case37 | Pneumocystis jirovecii | Pneumocystis jirovecii__38564 |
| case38 | Pneumocystis jirovecii | Staphylococcus epidermidis__2430 Pneumocystis jirovecii__74675 Human herpesvirus 4__8869 Human herpesvirus 7__8539 Human herpesvirus 5__3305 |
| case92 | Aspergillus fumigatus  Influenza A virus | Aspergillus fumigatus__989 Influenza A virus__182321 |
| case40 | （-） | GB virus C__18527 Human herpesvirus 7__11680 Human herpesvirus 1__623 Streptococcus mitis__2500 streptococcus constellatus__1824 Pneumocystis jirovecii__1 |
| case41 | Pneumocystis jirovecii SARS-CoV-2 | Pneumocystis jirovecii__1166119 Candida albicans__1966 SARS-CoV-2__71158 Human polyomavirus 5__595 Human parvovirus B19__117 |
| case42 | Influenza A virus | Influenza A virus__585544 |
| case43 | Influenza A virus | Influenza A virus__6288 |
| case44 | Acinetobacter baumannii Pneumocystis jirovecii  Influenza A virus | Acinetobacter baumannii__552 Streptococcus oralis__1623 Pneumocystis jirovecii__19873 Influenza A virus__283 |
| case45 | Klebsiella pneumoniae | Klebsiella pneumoniae__661515 Tropheryma whipplei__556 |
| case46 | Pneumocystis jirovecii | Tropheryma whipplei__632 streptococcus constellatus__15086 Staphylococcus hominis__2515 Haemophilus parainfluenzae__1566 Pneumocystis jirovecii__3123 Human rhinovirus C__332 Human herpesvirus 5__9519 Human herpesvirus 7__10660 |
| case47 | Pneumocystis jirovecii  Haemophilus influenzae | Haemophilus influenzae__130386 Pneumocystis jirovecii__20162 Human rhinovirus B__427 Human herpesvirus 4__372502 Human herpesvirus 74869 Human herpesvirus 5__170 |
| case48 | Pseudomonas aeruginosa  Pneumocystis jirovecii  Aspergillus fumigatus | Moraxella catarrhalis__810385 Pseudomonas aeruginosa__2464 Streptococcus mitis__1804 Pneumocystis jirovecii__10370 Aspergillus fumigatus__220 Human herpesvirus 7__2125 |
| case49 | （-） | Streptococcus mitis__1053 Human herpesvirus 7__41274 Human herpesvirus 4__4046 |
| case50 | Pneumocystis jirovecii  Haemophilus influenzae | Haemophilus influenzae__1541405 Streptococcus mitis__34492 Streptococcus anginosus__7155 Streptococcus constellatus__4891 Pneumocystis jirovecii__11754 Human herpesvirus 7__1726 |
| case51 | （-） | Streptococcus oralis__5391 Streptococcus anginosus__3485 Staphylococcus hominis__1185 Human herpesvirus 7__20070 Human herpesvirus 4__2498 |
| case52 | Aspergillus flavus  Pneumocystis jirovecii | Aspergillus flavus__248 Pneumocystis jirovecii__1444 Human herpesvirus 7__155311 |
| case53 | （-） | Streptococcus mitis__73884 Haemophilus parainfluenzae__1076 Human polyomavirus 5__892 |
| case54 | Mycoplasma pneumoniae  Influenza B virus | Mycoplasma pneumoniae__124358 Streptococcus mitis__7976 Influenza B virus__4375 |
| case55 | Escherichia coli  Klebsiella pneumoniae | Escherichia coli__498906 Klebsiella pneumoniae__343907 Ligilactobacillus salivarius__39278 Streptococcus anginosus__31017 Streptococcus oralis__6350 Staphylococcus epidermidis__5189 Brevundimonas diminuta__1287 Clavispora lusitaniae__15122 Nakaseomyces glabrata__312 Candida tropicalis__19 Human herpesvirus 4__865 |
| case56 | Aspergillus niger  Influenza A virus | Streptococcus mitis__29375 Staphylococcus epidermidis__8466 Corynebacterium striatum__2627 Haemophilus parainfluenzae__1085 Aspergillus niger__3394 Human adenovirus type 3__9485 Influenza A virus__182263 Human herpesvirus 7__36610 |
| case57 | Aspergillus niger | Streptococcus mitis__139670 Streptococcus anginosus__22355 streptococcus constellatus__14935 Streptococcus oralis__14807 Staphylococcus epidermidis__7977 Abiotrophia defectiva__3892 Haemophilus parainfluenzae__2220 Aspergillus niger__5382 Corynebacterium striatum__1050 Nakaseomyces glabrata__11530 Human herpesvirus 7__164560 Human herpesvirus 4__16189 |
| case58 | Pseudomonas aeruginosa Klebsiella pneumoniae  Influenza A virus | Pseudomonas aeruginosa__8131 Klebsiella pneumoniae__2529 streptococcus constellatus__1184 Influenza A virus__1723219 Human parainfluenza virus type 1__181 |
| case59 | （-） | （-） |
| case68 | Pneumocystis jirovecii | Tropheryma whipplei__15910 Staphylococcus epidermidis__275427 Streptococcus oralis__5818 Haemophilus parainfluenzae__196 Pneumocystis jirovecii__5326 Candida albicans__244 Human herpesvirus 7__14965 Human herpesvirus 5__830 |
| case61 | （-） | Moraxella catarrhalis__832 Human herpesvirus 1__4700 Human herpesvirus 5__1312 |
| case62 | Pneumocystis jirovecii | Streptococcus oralis__74172 Streptococcus mitis__54312 Ligilactobacillus salivarius__46219 Haemophilus parainfluenzae__5486 Corynebacterium striatum__1131 Pneumocystis jirovecii__336212 Human herpesvirus 7__711119 |
| case63 | Escherichia coli | Escherichia coli__836 Staphylococcus epidermidis__10459 |
| case64 | Pneumocystis jirovecii SARS-CoV-2 | Ureaplasma urealyticum__158 Pneumocystis jirovecii__15191 SARS-CoV-2__836831 |
| case65 | Enterococcus faecium | Enterococcus faecium__8516 Candida albicans__25366 Human respiratory syncytial virus subgroup B__591807 |
| case66 | （-） | Human rhinovirus A__1023692 Streptococcus mitis__14143 Streptococcus oralis__12590 Staphylococcus epidermidis__2873 |
| case67 | Enterococcus faecalis | Enterococcus faecalis__283386 Human respiratory syncytial virus subgroup B__119942 Streptococcus mitis__108321 Brevundimonas diminuta__2607 Ligilactobacillus salivarius__2565 Staphylococcus hominis__644 Fusobacterium nucleatum__122 |
| case39 | Aspergillus fumigatus | Aspergillus fumigatus__120 Human herpesvirus 7__17064 Human herpesvirus 4__7406 Streptococcus mitis__7163 |
| case69 | Staphylococcus epidermidis | Candida albicans__16256 Human herpesvirus 7__5815 Staphylococcus epidermidis__352873 Streptococcus mitis__44592 |
| case70 | Aspergillus terreus  SARS-CoV-2  Influenza B virus | Aspergillus terreus__442 SARS-CoV-2__1173221 Influenza B virus__70612 Human herpesvirus 4__1216 Human herpesvirus 7__57704 Staphylococcus epidermidis__5404 Streptococcus mitis__4684 Streptococcus anginosus__3129 Streptococcus oralis__2772 Ligilactobacillus salivarius__2318 |
| case71 | SARS-CoV-2  Influenza B virus | Candida tropicalis__1383 SARS-CoV-2__52730 Influenza B virus__12883 Human herpesvirus 7__3956 Human herpesvirus 5__1678 |
| case72 | Human respiratory syncytial virus subgroup B Influenza B virus | Human respiratory syncytial virus subgroup B__294769 Influenza B virus__5255 Human herpesvirus 7__7945 Human herpesvirus 4__3257 Streptococcus mitis__46346 Staphylococcus epidermidis__11769 Haemophilus parainfluenzae__1082 |
| case73 | Pseudomonas aeruginosa Influenza B virus  Human herpesvirus 5 | Pseudomonas aeruginosa__53397 Influenza B virus__78153 Human herpesvirus 5__53784 Human herpesvirus 7__88074 Human herpesvirus 4__7292 Streptococcus mitis__267982 |
| case74 | Enterococcus faecalis  Pneumocystis jirovecii  SARS-CoV-2 | Enterococcus faecalis__587470 Pneumocystis jirovecii__35368 SARS-CoV-2__427549 Human herpesvirus 7__90525 Human herpesvirus 5__7555 Human herpesvirus 4__5284 Ligilactobacillus salivarius__77414 |
| case75 | Acinetobacter baumannii | Acinetobacter baumannii__1282543 Human herpesvirus 1__50817 Human herpesvirus 5__15949 Human herpesvirus 4__1498 Meyerozyma guilliermondii__1068 |
| case76 | Pneumocystis jirovecii  SARS-CoV-2 | Acinetobacter pittii__2367 Enterococcus faecium__1030 Burkholderia multivorans__137 Pneumocystis jirovecii__761868 SARS-CoV-2__611895 Human parvovirus B19__1091 Human herpesvirus 4__312157 Human herpesvirus 1__9090 Human herpesvirus 7__2464 Human herpesvirus 5__1528 Staphylococcus epidermidis__12773 Haemophilus parainfluenzae__8211 Streptococcus oralis__5323 |
| case77 | Haemophilus influenzae | Haemophilus influenzae__414052 Moraxella catarrhalis__12987 Human herpesvirus 5__454 Human herpesvirus 4__9475 Streptococcus mitis__46110 |
| case78 | Mycobacterium tuberculosis complex | Mycobacterium tuberculosis complex __41034 Human herpesvirus 4__43195 Streptococcus mitis__19518 |
| case79 | Pneumocystis jirovecii | Pneumocystis jirovecii__766906 Candida albicans__4042 Human rhinovirus A__21932 Streptococcus anginosus__3302 Streptococcus mitis_1521 |
| case80 | SARS-CoV-2 | Candida albicans__83 SARS-CoV-2__840619 Human herpesvirus 5__383 Human herpesvirus 1__354 |
| case81 | Moraxella catarrhalis  Escherichia coli | Moraxella catarrhalis__526044 Escherichia coli__2706 |
| case82 | Moraxella catarrhalis | Moraxella catarrhalis__663581 Human herpesvirus 7__1544 Streptococcus mitis__1251 |
| case83 | （-） | Human herpesvirus 7__45957 Human herpesvirus 4__829 Brevundimonas diminuta__137734 Staphylococcus epidermidis__52156 Haemophilus parainfluenzae__22629 Streptococcus mitis__22211 |
| case84 | Enterococcus faecalis | Enterococcus faecalis__145009 Human respiratory syncytial virus subgroup B__4572 Human herpesvirus 1__164567 Human herpesvirus 4__16237 Human herpesvirus 7__3772 Ligilactobacillus salivarius__201153 Streptococcus mitis__197398 |
| case85 | Haemophilus influenzae Streptococcus pneumoniae | Haemophilus influenzae__296497 Streptococcus pneumoniae__170136 Human herpesvirus 7__1458 Human herpesvirus 4__1649 Ligilactobacillus salivarius__2526 |
| case86 | SARS-CoV-2 | SARS-CoV-2__413095 Streptococcus mitis__4138 |
| case87 | （-） | （-） |
| case88 | （-） | （-） |
| case89 | （-） | （-） |
| case90 | （-） | （-） |
| case91 | （-） | （-） |
| case106 | Acinetobacter baumannii | （-） |
| case93 | Pneumocystis jirovecii | Pneumocystis jirovecii__8379 Human herpesvirus 7__24684 streptococcus constellatus__7687 Staphylococcus epidermidis__2918 Haemophilus haemolyticus__1260 |
| case94 | Haemophilus influenzae | Haemophilus influenzae__175525 Streptococcus mitis__2393 |
| case95 | Mycobacterium tuberculosis complex | Mycobacterium tuberculosis complex __25061 Streptococcus mitis__11112 Streptococcus oralis__1805 Staphylococcus hominis__1558 Haemophilus parainfluenzae__520 |
| case96 | Acinetobacter baumannii | Acinetobacter baumannii__7104 Streptococcus anginosus__4978 Streptococcus oralis__3686 |
| case97 | Haemophilus influenzae  Acinetobacter baumannii | Haemophilus influenzae__173081 Brevundimonas diminuta__4546003 Streptococcus anginosus__2165115 Staphylococcus epidermidis__1737 Human herpesvirus 7__10409 Human herpesvirus 1__3726 Human herpesvirus 4__904 Human herpesvirus 5__823 |
| case98 | Haemophilus influenzae  SARS-CoV-2 | Haemophilus influenzae__2319 SARS-CoV-2__19 |
| case99 | Mycobacterium tuberculosis complex | Mycobacterium tuberculosis complex __16 |
| case100 | Pneumocystis jirovecii  SARS-CoV-2 | Pneumocystis jirovecii__4236 SARS-CoV-2__25970 |
| case101 | Enterococcus faecium  Mycobacterium tuberculosis complex  Aspergillus fumigatus | Enterococcus faecium__2386 Mycobacterium tuberculosis complex __141 Clavispora lusitaniae__1013 Aspergillus fumigatus__1134 |
| case102 | （-） | （-） |
| case103 | Moraxella catarrhalis | Moraxella catarrhalis__224645 Exophiala dermatitidis__4 Streptococcus oralis__65139 Brevundimonas diminuta__489 Haemophilus parainfluenzae__267 Veillonella parvula__163 Candida krusei__6102 Candida albicans__2486 Human herpesvirus 4__752 Human herpesvirus 1__131 |
| case104 | Enterococcus faecium | Enterococcus faecium__3198 Streptococcus mitis__281 Human herpesvirus 5__66 Human herpesvirus 4__29 Haemophilus parainfluenzae__10 |
| case105 | Pseudomonas aeruginosa Aspergillus fumigatus | Pseudomonas aeruginosa__983 Aspergillus fumigatus__8431 Ligilactobacillus salivarius__9 Human rhinovirus C__466 Human herpesvirus 4__27 Human herpesvirus 1__14 |
| case60 | Haemophilus influenzae | Haemophilus influenzae__2431 Streptococcus mitis__53 Abiotrophia defectiva__6 Veillonella parvula__3 Candida albicans__896 Human herpesvirus 1__168 Human herpesvirus 4__12 |
| case107 | Haemophilus influenzae | Streptococcus agalactiae__140 Streptococcus anginosus__466 Haemophilus influenzae__1959 Abiotrophia defectiva__9 Veillonella parvula__5 |
| case108 | Haemophilus parainfluenzae  Pneumocystis jirovecii | Streptococcus pneumoniae__105 Haemophilus parainfluenzae__1887 Streptococcus mitis__1250 Streptococcus oralis__452 Ligilactobacillus salivarius__144 Candida albicans__22 Pneumocystis jirovecii__4233 Human herpesvirus 4__375 |
| case109 | Haemophilus parainfluenzae | Haemophilus parainfluenzae__6913 Streptococcus mitis__1148 streptococcus constellatus__911 Brevundimonas diminuta__185 Veillonella parvula__41 Fusobacterium nucleatum__5 Candida albicans__40 Human herpesvirus 4__42 Human herpesvirus 7__4 |
| case110 | （-） | Streptococcus anginosus__13 streptococcus constellatus__8 Human herpesvirus 4__33 |
| case111 | Klebsiella pneumoniae | Klebsiella pneumoniae__2926 Haemophilus parainfluenzae__37 Candida albicans__3 |
| case112 | Klebsiella pneumoniae Pneumocystis jirovecii | Klebsiella pneumoniae__103288 Haemophilus influenzae__74 Escherichia coli__34248 Ligilactobacillus salivarius__28 Streptococcus mitis__4 Pneumocystis jirovecii__5782 Human parainfluenza virus type 3__36783 Human herpesvirus 4__191 |
| case113 | Aspergillus fumigatus | Aspergillus fumigatus__781 Human herpesvirus 6B__5 |
| case114 | Pneumocystis jirovecii | Pneumocystis jirovecii__4850 |
| case115 | Klebsiella pneumoniae  Enterococcus faecalis | Streptococcus pneumoniae__377 Klebsiella pneumoniae__234510 Enterococcus faecalis__109 Streptococcus oralis__1296 Streptococcus mitis__659 Veillonella parvula__83 Lacticaseibacillus rhamnosus__40 Candida parapsilosis__333 Human herpesvirus 4__45 |
