# Supplementary material for: Clinical features, pathogens, and prognosis of immunocompromised host pneumonia in patients with malignancies
Source: Front Cell Infect Microbiol. 2025 Nov 18;15:1646513. doi: 10.3389/fcimb.2025.1646513 (PMC12669106; doi:10.3389/fcimb.2025.1646513)
Supplement: Supplementary Table 3 — Comparison between patients with pathogen negative and pathogen positive. [file Table3.docx]

|  | Pathogen negative | Pathogen positive | P |
| --- | --- | --- | --- |
| ICU admission | 4/23  (17.4%) | 30/92  (32.6%) | 0.153 |
| ICU mortality | 1/4  (25.0%) | 24/30  (80%) | 0.048 |
| 28-day mortality | 1/23  (4.3%) | 31/92  (33.7%) | 0.005 |
| Vasoactive drugs | 1/23  (4.3%) | 17/92  (18.5%) | 0.178 |
| IMV | 1/23  (4.3%) | 30/92  (32.6%) | 0.027 |

**Supplementary Table S3.** Comparison between patients with pathogen negative and pathogen positive.
