# Supplementary material for: Clinical features, pathogens, and prognosis of immunocompromised host pneumonia in patients with malignancies
Source: Front Cell Infect Microbiol. 2025 Nov 18;15:1646513. doi: 10.3389/fcimb.2025.1646513 (PMC12669106; doi:10.3389/fcimb.2025.1646513)
Supplement: Supplementary Table 4 — Comparison between patients with Single pathogen infection and Coinfected. [file Table4.docx]

|  | Single pathogen infection | Coinfected | P |
| --- | --- | --- | --- |
| ICU admission | 13/50 (26.0%) | 17/42 (40.5%) | 0.140 |
| ICU mortality | 10/13 (76.9%) | 14/17 (82.4%) | 1 |
| 28-day mortality | 14/50 (28.0%) | 17/42 (40.5%) | 0.207 |
| Vasoactive drugs | 8/50 (16.0%) | 9/42 (21.4%) | 0.504 |
| IMV | 11/50 (22.0%) | 15/42 (35.7%) | 0.428 |

**Supplementary Table S4.** Comparison between patients with Single pathogen infection and Coinfected.
